# No difference in striatal dopamine transporter availability between active smokers, ex-smokers and non-smokers using [^123^I]FP-CIT (DaTSCAN) and SPECT

**DOI:** 10.1186/2191-219X-3-39

**Published:** 2013-05-20

**Authors:** Gerda Thomsen, Gitte Moos Knudsen, Peter S Jensen, Morten Ziebell, Klaus K Holst, Susanne Asenbaum, Jan Booij, Jacques Darcourt, John C Dickson, Özlem L Kapucu, Flavio Nobili, Osama Sabri, Terez Sera, Klaus Tatsch, Livia Tossici-Bolt, Koen Van Laere, Thierry Vander Borght, Andrea Varrone, Marco Pagani, Lars Hageman Pinborg

**Affiliations:** 1 Neurobiology Research Unit 9201, Rigshospitalet and Copenhagen University Hospital, Blegdamsvej 9, Copenhagen 2100, Denmark; 2 Department of Nuclear Medicine, Medical University of Vienna, Vienna 1090, Austria; 3 Department of Nuclear Medicine, Academic Medical Centre University of Amsterdam, Amsterdam 1100, The Netherlands; 4 Nuclear Medicine Department, Centre Antoine Lacassagne, University of Nice-Sophia Antipolis, Nice 06189, France; 5 Institute of Nuclear Medicine, UCLH NHS Foundation Trust and University College, London NW12BU, UK; 6 Department of Nuclear Medicine, Gazi University, Faculty of Medicine, Ankara 06500, Turkey; 7 Clinical Neurophysiology Unit, Department of Neurosceince, Ophthalmology and Genetics, University of Genoa, Genoa 16132, Italy; 8 Department of Nuclear Medicine, University of Leipzig, Leipzig 04103, Germany; 9 Department of Nuclear Medicine and Euromedic Szeged, University of Szeged, Szeged 6720, Hungary; 10 Department of Nuclear Medicine, Municipal Hospital of Karlsruhe Inc., Karlsruhe 76133, Germany; 11 Department of Medical Physics and Bioengineering, University Hospital Southampton NHS Foundation Trust, Southampton SO166YD, UK; 12 Nuclear Medicine, University Hospital and KU Leuven, Leuven 3000, Belgium; 13 Nuclear Medicine Division, Mont-Godinne Medical Center, Université Catholique de Louvain, Louvain-la-Neuve, Yvoir 5530, Belgium; 14 Department of Clinical Neuroscience, Karolinska University Hospital, Stockholm 17176, Sweden; 15 Department of Nuclear Medicine, Karolinska University Hospital, Stockholm 17176, Sweden; 16 Epilepsy Clinic 8501, Rigshospitalet and Copenhagen University Hospital, Copenhagen 2100, Denmark; 17 Department of Biostatistics, University of Copenhagen, Copenhagen 2100, Denmark

**Keywords:** Tobacco smoking, Non-smoking, SPECT, [^123^I]FP-CIT (DaTSCAN), Dopamine transporter

## Abstract

**Background:**

Mesolimbic and nigrostriatal dopaminergic pathways play important roles in both the rewarding and conditioning effects of drugs. The dopamine transporter (DAT) is of central importance in regulating dopaminergic neurotransmission and in particular in activating the striatal D_2_-like receptors. Molecular imaging studies of the relationship between DAT availability/dopamine synthesis capacity and active cigarette smoking have shown conflicting results. Through the collaboration between 13 SPECT centres located in 10 different European countries, a database of FP-CIT-binding in healthy controls was established. We used the database to test the hypothesis that striatal DAT availability is changed in active smokers compared to non-smokers and ex-smokers.

**Methods:**

A total of 129 healthy volunteers were included. Subjects were divided into three categories according to past and present tobacco smoking: (1) non-smokers (*n* = 64), (2) ex-smokers (*n* = 39) and (3) active smokers (*n* = 26). For imaging of the DAT availability, we used [^123^I]FP-CIT (DaTSCAN) and single photon emission computed tomography (SPECT). Data were collected in collaboration between 13 SPECT centres located in 10 different European countries. The striatal measure of DAT availability was analyzed in a multiple regression model with age, SPECT centre and smoking as predictor.

**Results:**

There was no statistically significant difference in DAT availability between the groups of active smokers, ex-smokers and non-smokers (*p* = 0.34). Further, we could not demonstrate a significant association between striatal DAT and the number of cigarettes per day or total lifetime cigarette packages in smokers and ex-smokers.

**Conclusion:**

Our results do not support the hypothesis that large differences in striatal DAT availability are present in smokers compared to ex-smokers and healthy volunteers with no history of smoking.

## Background

The behavioural and neurobiological effects of smoking are similar to those of other addictive substances [1], and several studies have demonstrated the involvement of the mesolimbic dopaminergic system in mediating the response to cigarette smoking or nicotine intake. Nicotine-induced dopamine release has been demonstrated in rodents [2-4] and non-human primates [5-8]. These findings have been indirectly supported by several positron emission tomography (PET) studies where decreases of [^11^C]raclopride binding (thought to reflect increases in the extracellular concentration of dopamine) in the ventral striatum/nucleus accumbens following smoking [6,9-12] or nicotine intake [13] were observed. PET-studies, using radiopharmaceuticals for the dopamine D_2/3_ receptors, have demonstrated significant associations between dopamine release and reduction in craving [9,10], enhancement of pleasure [14,15] and the severity of nicotine dependence [12]. However, the reduction in D_2_/_3_ binding upon smoking compared to the baseline condition is modest (5% to 10%) in these studies compared to that following cocaine (20% to 30%) [16] and amphetamine [17-19]. Such an effect of 5% to 10% is similar to the test-retest variability of molecular imaging techniques using PET and SPECT [20-22] and may be difficult to demonstrate in PET and SPECT studies using small data samples. This may explain the failure of some PET studies to demonstrate changes in [^11^C]raclopride binding upon nicotine administration [14,15]. Dopamine release in the ventral striatum/nucleus accumbens has been demonstrated to be directly mediated through the binding of nicotine to α4β2 nAChRs leading to an increase in firing rate in dopamine neurons of the ventral tegmental area [23,24]. Previous human imaging studies do not provide strong support for the idea that postsynaptic dopamine D_2_/_3_ receptor availability is affected by chronic exposure to cigarette smoke. A reduced dopamine D_2_/_3_ receptor availability was demonstrated in the putamen in men [25], but not in woman [26]. Two SPECT studies failed to demonstrate changes in striatal dopamine D_2_/_3_ receptor availability in smokers compared to non-smokers [27,28].

The dopamine transporter (DAT) provides the primary mechanism through which dopamine is cleared from the extracellular fluid after its release from the presynaptic cell. However, only few studies have addressed the effect of chronic dosing of nicotine on the DAT [29]. Nicotine is not a competitor nor a substrate for DAT [30], and it does not bind to a site on the DAT protein [31]. Nicotine appears to induce changes in DAT function by indirect mechanisms which include both augmentation (enhancing amphetamine-induced reverse transport of dopamine by DAT) and reduction (increase in cell surface DAT expression) of dopaminergic neurotransmission [32]. Human imaging studies of DAT availability in relation to chronic tobacco smoking are sparse and have generated conflicting results [28,33] possibly related to methodological problems including the choice of radioligand and sample size. We studied the DAT availability using [^123^I]FP-CIT-SPECT in a group of 26 active smokers, 39 ex-smokers and 64 subjects with no history of smoking. We tested the hypothesis that DAT availability is changed in active smokers compared to ex-smokers and subjects with no history of tobacco smoking. Understanding the mechanisms underlying the neurobiological effects of nicotine on the regulation of DAT may have the potential to translate into new and possibly individualized treatment strategies.

## Methods

### Participants

The European Normal Control Database of DaTSCAN (ENCDAT) study is an initiative taken by the Neuroimaging Committee of the European Association of Nuclear Medicine (EANM). The database was established through the collaboration between 13 SPECT centres located in 10 different European countries. The centres were selected by EANM based on their involvement in SPECT imaging of the dopaminergic system and their high level of experience and quality of brain SPECT imaging. The protocol was approved by the medical ethical committees of all participating centers and was performed in accordance with the ethical standards of the Declaration of Helsinki. All subjects gave written informed consent to participate in the study.

In this study, 129 of the available ENCDAT healthy volunteers (all Caucasian) were included. Twelve healthy volunteers had to be excluded because no imaging data for the scatter windows data were available, and ten healthy volunteers (ex-smokers) had to be excluded because of lifetime usage of less than 60 packages of cigarettes. Our sample had a balanced male-to-female ratio (70 males and 59 females), and the age range was 20 to 83 years (>20 per decade, expected for ages between 80 to 90 years). The distribution between the participating centres was as follows: Amsterdam (*n* = 9), Ankara (*n* = 10), Copenhagen (*n* = 13), Genoa (*n* = 14), Leipzig (*n* = 13), Leuven (*n* = 16), London (*n* = 10), Munich (*n* = 11), Nice (*n* = 4), Southampton (*n* = 3), Stockholm (*n* = 13) and Yvoir (*n* = 13).

Inclusion criteria were the following:

• No history of parkinsonism in first-degree relatives

• No medication known to affect DAT binding

• Absence of psychiatric symptoms as evaluated by the following: Symptom Checklist-90-R score < 63, Beck Depression Inventory score ≤ 9 and Mini-Mental State Examination ≥ 28

• Negative urine screening for drugs (ten drugs)

• Body temperature ≤ 38.5°C on the day of scanning

• Negative pregnancy test in premenopausal females

### Smoking data

All subjects were interviewed about tobacco smoking habits (as part of the inclusion criteria) and were quantified through The Copenhagen Smoking Questionnaire [34]. Data regarding the time of the last cigarette before scanning was not available. Subjects were divided into three categories according to past and present tobacco smoking habits: (1) non-smokers (*n* = 64), (2) ex-smokers (more than 60 total numbers of packages in lifetime, *n* = 39) and (3) active smokers (1 to 30 cigarettes per day, *n* = 26), as shown in Table 1. Ten of the active smokers used more than 15 cigarettes per day. For smokers, the cigarette use per day was registered, and for all smokers (active smokers and ex-smokers), the total number of packages in lifetime was registered.

**Table 1:** Age and smoking habits for 129 healthy volunteers

	**Number**	**Mean age ± SD (range) in years**	**Current average number of cigarettes per day ± SD (range)**	**Total number of packages in lifetime mean (range)**
Non-smokers	64 (34 males)	51.7 ± 18.5 (20 to 81)	0	0
Ex-smokers	39 (21 males)	59.2 ± 15.4 (25 to 83)	0	4,497 (66 to 28,220)
Active smokers	26 (15 males)	47.2 ± 19.4 (21 to 79)	11.2 ± 8.6 (1 to 30)	5,991 (60 to 24,630)

### Data acquisition and reconstruction

Data acquisition was performed according to the ENCDAT protocol (EANM Research Ltd. (EARL)/European Network of Excellence for Brain Imaging) using the scanners and collimators specified in the ENCDAT protocol. All camera systems had passed elaborate quality control and phantom measurements [35]. SPECT acquisition was started 3 to 4 h after an average intravenous bolus of 180.5 MBq (range 152 to 215 MBq) of [^123^I]FP-CIT (GE Healthcare, Amersham, UK). Image reconstruction was carried out at a core centre using the HERMES HOSEM software (HERMES Medical Solutions, Stockholm, Sweden), using iterative reconstruction with 10 subsets and 10 iterations for 120 projections and 8 subsets and 12 iterations for 128 projections to give a similar number of EM equivalent iterations [36]. Reconstructions were performed with attenuation and scatter corrections using the triple-energy window method. After reconstruction, images were smoothed on the HERMES workstation with a 3D Butterworth filter (cut-off 1.2 cm^–1^, order 10).

### ROI delineation and SBR calculation

**Figure 1:**
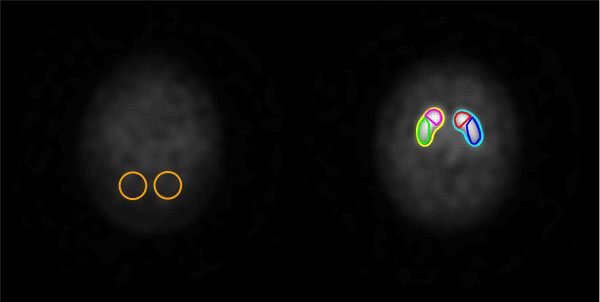
**Two horizontal slices from the constructed [**^**123**^**I]-FP-CIT template.** Illustrating exact position and configuration of the reference (left) and striatal (right) ROIs.

We used the ratio of specifically bound radioligand to that of nondisplaceable radioligand in tissue (specific binding ratio (SBR)) calculated between 3 and 4 h after tracer injection as a measure of the DAT availability [37]. Regions of interest (ROIs) were delineated, and SBR was calculated using *DATquan* (Figure 1) [38]. DATquan offers a fast, accurate, and highly reproducible method for semi-automatic VOI delineation using a template-based approach.

## Results

For the study of the association between DAT measurements and smoking habits, we used *a priori* a linear model where we adjusted for age, which has previously been identified as an important confounder. Data were collected from several different centers, and to account for potential cluster effects, we therefore used a random intercept model.

In general, similar results were obtained from a more parsimonious model for the clustering based on marginal models with robust standard errors (i.e., a generalized estimating equation framework). Here, we only report results (maximum likelihood estimates) from the random intercept model.

Variance homogeneity of residuals across the different centers was tested using a likelihood ratio test which indicated that this assumption was reasonable (*p* = 0.77). In all cases, residual analysis revealed that a log transformation of the DAT measurements was favorable (all results based on the log transformation and hence parameters can be interpreted as log relative differences). However, quite similar conclusions were reached based on modeling of the data on their original scale. Linearity of the continuous predictor age was assessed using a linear mixed additive model and by inclusion of polynomial terms in the model. R version 2.15 was used for all analyses [39], http://www.R-project.org/).

**Figure 2:**
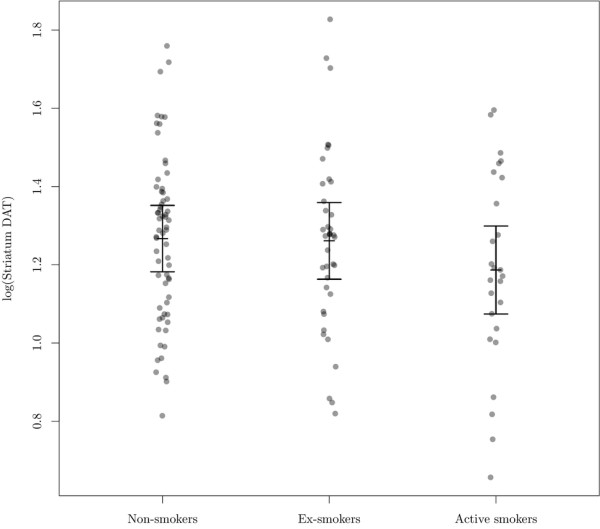
**Association between striatal DAT availability and smoking status.** The vertical bars shows the estimated mean within each group with 95% confidence limits as estimated by a random intercept model (with a variance component defined by the centre) adjusting for age at scan (reference, mean age = 53 years). Individual points are partial residuals (i.e., best linear unbiased predictor of the residuals plus the estimated intercept within each group).

Active smoking was not associated with any statistically significant effects on the striatal DAT availability compared to non-smokers (7.7% lower DAT availability, 95% confidence limits (−17.3%, 3.0%), *p* = 0.15); the same was true for DAT availability in caudate nucleus (95% confidence limits (−16.7%, 3.2%), *p* = 0.17) and putamen (95% confidence limits (−17.5%, 3.9%), *p* = 0.19). There was no statistically significant difference in DAT availability between the groups of active smokers, ex-smokers and non-smokers (*p* = 0.34) as seen in Figure 2.

There was a clear effect of age on DAT availability with an age decline in striatum equal to 4.6% per decade (95% confidence limits (−6.9%, −2.2%)), in line with a previous study [40]. The estimated residual standard deviation within clusters was 0.228, and the standard deviation of the random intercept (between clusters) was 0.115.

**Figure 3:**
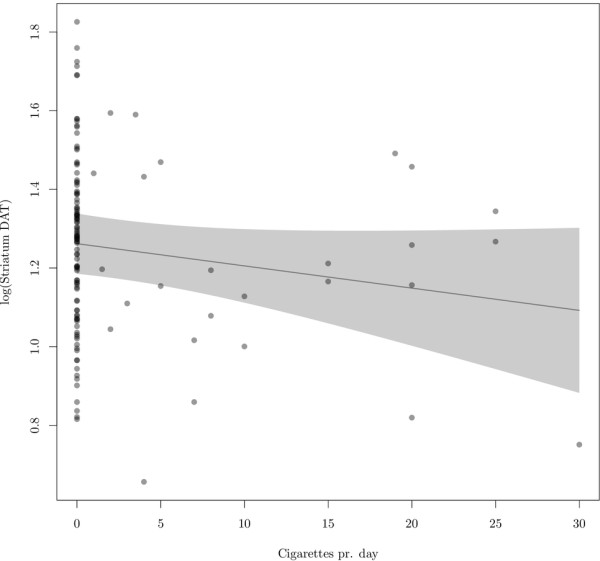
**Estimated association between log (striatal DAT availability) and number of cigarettes per day (currently).** With 95% pointwise confidence limits as estimated by a random intercept model adjusting for age (reference, mean age = 53 years). Individual points are the partial residuals, defined as the best linear unbiased predictor of the residuals plus the estimated cigarettes per day effect.

As seen in Figure 3, there was no statistically significant association between striatal DAT availability and numbers of cigarettes per day (in smokers) with an estimated 5.6% decrease in DAT availability per ten cigarettes per day ((−12.6%, 1.3%), *p* = 0.114); furthermore, we found no negative correlation between total cigarette packages in lifetimes and striatal DAT availability (0.6% decrease in DAT availability per 1,000 packages, 95% confidence limits (1.4%, 0.3%), *p* = 0.20) in caudate nucleus (*p* = 0.21) and putamen (*p* = 0.19). There was no statistically significant difference in striatal DAT availability (*p* = 0.906) in ex-smokers compared to non-smokers and in ex-smokers compared to active smokers (*p* = 0.152).

## Discussion

In this large sample, we did not observe any statistically significant effect of tobacco smoking on striatal DAT availability as measured with [^123^I]FP-CIT SPECT in active smokers (*n* = 26) and ex-smokers (*n* = 39) compared to non-smokers (*n* = 64). In addition, a statistically significant association between striatal DAT availability and numbers of cigarettes per day or total cigarette packages in lifetimes was not found. Our results in active smokers compared to ex-smokers do not support the idea of changed striatal DAT availability upon daily nicotine administration. However, the possible acute effect of smoking on striatal DAT availability still needs to be explored.

This study is in line with the results of Staley et al. which did not demonstrate changes in striatal [^123^I]β-CIT binding in 21 smokers compared to 21 non-smokers [41]. [^123^I]β-CIT binds to both the striatal DAT and the serotonin transporter (SERT), and the non-selectivity of this tracer hampers the conclusions to be drawn from this study on the relative role of DAT compared to SERT in smoking. A recent study by Erritzoe et al. also failed to demonstrate significant differences in the binding of the PET tracer [^11^C]DASB to SERT in smokers compared to non-smokers [34]. Our study does not replicate the findings of a decreased striatal DAT availability in smokers [28,33] using [^99m^Tc]TRODAT-1 SPECT. We believe that the discrepancies between the findings in the two [^99m^Tc]TRODAT-1 SPECT studies and the results of our study and the study of Staley et al. [41] are related to the sample size (8 and 11 active smokers were included in the [^99m^Tc]TRODAT-1 SPECT studies) and possibly the imaging properties of [^99m^Tc]TRODAT-1 related to a small ratio between binding of tracer in striatum compared to the reference region leading to data being more susceptible to noise. In a [^18^F]fluorodopa PET of the striatal presynaptic dopamine activity in active smokers (*n* = 9) compared to non-smokers (*n* = 10), a significantly higher uptake of striatal [^18^F]fluorodopa was demonstrated in smokers compared to non-smokers [42]. A higher uptake of [^18^F]fluorodopa is generally interpreted as a result of an increased dopamine synthesis capacity. However, a higher uptake of [^18^F]fluorodopa may be tracing several independent processes including BBB transport, competition with other amino acids for transport, uptake into neurons, decarboxylation to fluorodopamine and trapping within neuronal vesicles [43]. Thus, in addition to the small sample of active smokers in the [^18^F]fluorodopa PET study, the outcome parameter is not directly comparable to studies using radiotracers binding to the DAT.

This study has some limitations. (1) As seen in Table 1, the numbers of cigarettes smoked per day range from 1 to 30 cigarettes, and 10 of the subjects smoked more than 15 cigarettes per day. Thus, the group of heavy smokers is small. However, the DAT availability in the group smoking more than 15 cigarettes a day was not significantly different from ex-smokers and non-smokers. Furthermore, a statistical significant association between cigarettes smoked per day and DAT availability was not demonstrated (Figure 3), and according to our data, we would expect a very small decrease in striatal DAT of 0.6% if an individual consumed an additional 1,000 packages. (2) We did not control for passive smoking. However, based upon our dose–response data, we find it unlikely that passive smoking results in detectable changes in DAT availability in any of the three groups. (3) Though our data sample is larger than the previous studies on DAT availability in smoking, the study is still underpowered to detect subtle changes in DAT availability related to its variation between subjects and a [^123^I]FP-CIT SPECT test-retest variability of approximately 10%. Though not statistically significant, a trend toward a decrease in DAT availability (5.6% decrease in DAT availability per ten cigarettes per day, *p* = 0.114) was found. Our study may be underpowered to demonstrate a small effect. (4) In this multi-centre study, information regarding time of last cigarette smoked before injection of the radiotracer and information regarding nicotine dependence, smoking urges and passive smoking was not available. Future studies must address the acute effects of cigarette smoking and thus explain whether DAT binding predisposes to current smoking or whether current smoking influences DAT binding. In contrast to the current study using cross-sectional design, future studies could benefit from a longitudinal design testing DAT availability in patients before and after smoking cessation.

## Conclusion

No statistically significant effect of chronic tobacco smoking on striatal DAT availability or changes in DAT availability in previous smokers compared to subjects with no history of smoking was seen with [^123^I]FP-CIT SPECT. With the limitations underlined in the discussion, our data do not suggest that changes in the dopaminergic system resulting from smoking and/or nicotine administration involve any regulatory changes in DAT. Further studies are needed to address whether the DAT availability is susceptible to acute smoking or nicotine administration.

## Competing interests

Prof. Jan Booij is a consultant for GE Healthcare. All other authors have no competing interests.

## Authors’ contributions

All authors contributed to the design of the ENCDAT study. The authors MZ, GMK, SA, JB, JD, JCD, OLK, FN, OS, KT, LTB, KVL, TVB and MP recruited the subjects, interviewed the subjects about tobacco smoking habits and acquired SPECT data on the subjects. GT, PSJ, MZ, KKH, JCD, TS, LTB, AV and LHP analyzed the data. GT and LHP drafted the manuscript. All authors read and approved the final manuscript.
